# When Hearing Is Tricky: Speech Processing Strategies in Prelingually Deafened Children and Adolescents with Cochlear Implants Having Good and Poor Speech Performance

**DOI:** 10.1371/journal.pone.0168655

**Published:** 2017-01-05

**Authors:** Magdalene Ortmann, Pienie Zwitserlood, Arne Knief, Johanna Baare, Stephanie Brinkheetker, Antoinette am Zehnhoff-Dinnesen, Christian Dobel

**Affiliations:** 1 Institute for Biomagnetism and Biosignalanalysis, Muenster University Hospital, Muenster, Germany; 2 Jean-Uhrmacher-Institute for Clinical ENT-Research, University of Cologne, Cologne, Germany; 3 Department of Psychology, University of Muenster, Muenster, Germany; 4 Department of Phoniatrics and Pedaudiology, Muenster University Hospital, Muenster, Germany; 5 Department of Otorhinolaryngology, Jena University Hospital, Jena, Germany; Sun Yat-Sen University, CHINA

## Abstract

Cochlear implants provide individuals who are deaf with access to speech. Although substantial advancements have been made by novel technologies, there still is high variability in language development during childhood, depending on adaptation and neural plasticity. These factors have often been investigated in the auditory domain, with the mismatch negativity as an index for sensory and phonological processing. Several studies have demonstrated that the MMN is an electrophysiological correlate for hearing improvement with cochlear implants. In this study, two groups of cochlear implant users, both with very good basic hearing abilities but with non-overlapping speech performance (very good or very poor speech performance), were matched according to device experience and age at implantation. We tested the perception of phonemes in the context of specific other phonemes from which they were very hard to discriminate (e.g., the vowels in /bu/ vs. /bo/). The most difficult pair was individually determined for each participant. Using behavioral measures, both cochlear implants groups performed worse than matched controls, and the good performers performed better than the poor performers. Cochlear implant groups and controls did not differ during time intervals typically used for the mismatch negativity, but earlier: source analyses revealed increased activity in the region of the right supramarginal gyrus (220–260 ms) in good performers. Poor performers showed increased activity in the left occipital cortex (220–290 ms), which may be an index for cross-modal perception. The time course and the neural generators differ from data from our earlier studies, in which the same phonemes were assessed in an easy-to-discriminate context. The results demonstrate that the groups used different language processing strategies, depending on the success of language development and the particular language context. Overall, our data emphasize the role of neural plasticity and use of adaptive strategies for successful language development with cochlear implants.

## Introduction

After their initial development in the 1950s by Djourno and Eryies [1], cochlear implants (CI) provide individuals who are deaf with access to spoken language. Young children who were born deaf or became deafened before language development started can–under optimal conditions- even develop language performance within the normal range, enabling them to communicate with people of typical hearing [2]. There is nevertheless a surprisingly strong variance in language development in these children. Even when controlling for device experience (number of years with auditory experience after CI surgery) and age at implantation, a large amount of variance remains unexplained [3–5]. In this study, we attempt to shed light on phoneme perception in difficult language contrasts in two groups of congenitally deaf or prelingually deafened CI users. Both groups have very good basic hearing abilities, but differ in their language perception performance. We were particularly interested in the neural correlates of processing difficult phoneme contrasts, and used the mismatch negativity (MMN) component from the EEG. Difficult contrasts (e.g., the vowels in /bu/ and /bo/) were not predefined, but rather separately established for each participant on the basis of behavioral measures. This research complements and contrasts with earlier data on easy-to-discriminate contrasts in the same population [6]. Before explaining the differences between the two studies, we will introduce the background and methods in more detail.

### Phoneme Discrimination and Language Development in CI Users and Healthy Controls

During the course of development of the human race several thousand languages came into existence that enable us to express our thoughts and ideas with high precision. Languages can be characterized by a specific phoneme repertoire which represent the building blocks for words that may sound very similar, but differ in their meaning. Therefore, not only for the perception of words, but also in a larger sentential context, correct identification of phonemes is of crucial importance. “*Peter*, *please start the fan*!*”* leads to other actions than “*Peter*, *please start the van*!*”*. Interestingly, while adults are unable to distinguish phonemes that do not belong to their language environment, human babies can easily differentiate all of the world’s phonemes [7–9]. At around six months of age phoneme perception is altered by the exposure to the baby’s language environment. At this age, identification of native-language phonemes becomes facilitated while detection of non-native phonemes declines [10,11]. Importantly, phoneme discrimination abilities tested at this age are not only a good predictor of language performance at the age of two [12] but also have been discussed as possible predictors of language dysfunctions such as Specific Language Impairement (SLI) [13] or other developmental linguistic impairments in children [14,15]. Children with cochlear implants often miss a great amount of hearing experience before implantation leading to less exposure to the phonemes of their native language. With strong relations between phonological awareness and higher order language abilities as word decoding, reading or communication mode [16–20], it is not surprising that prelingually deafened CI users with very good or poor language development do also show strong differences in phoneme discrimination [6].

Still, the development of novel technologies, as e.g. better sound coding strategies implemented to the speech processors, improved the development of hearing after implantation to a substantial degree. Next to these technical improvements, there is increasing evidence that learning constitutes a crucial component for a successful outcome. In the elderly, hearing and especially language perception is often not good after initial activation of the implant, but improves quickly within 10 to 20 weeks [21]. Obviously, a period of adaptation or adjustment is needed, which may more appropriately be called ‘learning’ when it requires an active engagement of the hearer with her acoustic environment (for a review, see [22]).

### Testing Phoneme Discrimination in CI Users and Healthy Controls with the Mismatch Negativity

Mechanisms of auditory sensory processing and the consequences of learning were often investigated electrophysiologically with the mismatch negativity component (MMN). This component rises to a relatively infrequent stimulus, the so-called deviant, that appears in a stream of frequent stimuli, the standards [23]. Whenever the neural response evoked by the deviant does not match the still-available memory trace formed by the preceding standards, the MMN is triggered [24]. There is now firm evidence that the MMN can be used to monitor even subtle phonological and phonetic differences between stimuli (e.g., [25,26]) and also inform about the time course and success of learning novel contrasts (e.g., [27]). In line with fMRI data [28], there is strong evidence that the MMN is not uniquely evoked in the bilateral auditory cortices, but also has prefrontal sources in both hemispheres [29–31], especially for language perception [32,33]. While typical peaks of the MMN arise 150 to 250 ms after change onset, recent studies have reported that deviant detection indicated by ERPs can already appear 20 to 40 ms after change onset (for a recent review, see [34]). It is argued on this basis that novelty detection is a crucial mechanism for the functional organization of the auditory system.

It is not surprising that the MMN was used to investigate auditory perception in persons with CIs. Several studies have demonstrated that the MMN is a good indicator of learning–as e.g. for the improvement of phoneme discrimination abilities after CI implantation [35], which correlates with language perception abilities [36]. For example, postlingually deafened CI users with good perceptual abilities after CI implantation revealed a MMN very similar to controls with normal hearing, while the MMN was absent in CI users with poor hearing abilities [37–39]. Note that these studies investigated language perception in postlingually deafened CI users and, due to equipment and methods, could not draw conclusions about the cortical generators of the MMN. Even though source localization of ERP components does not provide the spatial accuracy of fMRI and PET, it is recommended for CI users because of strong safety concerns with fMRI (due to the metallic and magnetic components of the implant) as well as the invasive character of PET. Source localization with distributed models for ERP data of CI users has been successfully used before [6,40] and is applied frequently to other areas of language processing (e.g. [41–43]).

A major advantage of the MMN for research with CI patients lies in the use of an identical stimulus as both standard and deviant, appearing in different runs. By subtracting brain responses to these physically identical stimuli, electrophysiological artifacts of the implant can be removed [6,44]. In the present study, we registered the MMN in response to a particular phoneme (the deviant) when they were hard to differentiate from the standard by implant users (e.g. /bu/ vs. */bo/*). Importantly, we have used the very same stimuli before, with standards from which the deviant is more easily differentiated (/bu/ vs. */ba/*, [6]): two groups of prelingually deafened CI users with equally good overall hearing abilities, but who developed either good or poor language performance after implantation, displayed a MMN, but with different cortical origins. Source localization revealed an larger MMN for good performers in the left fronto-temporal cortex from 155 to 225 ms, emphasizing the crucial role of the frontal cortex in language perception [45]. This was followed by larger MMN for poor than for good performers, in the left auditory cortex, from 225 to 250 ms. Both groups were individually matched for age at implantation and device experience so that differences between groups could not be attributed to these factors.

Including our own study, research to date has focused on stimulus material that is easy to process for CI users. As a consequence, no differences were observed between good performers and controls with typical hearing [37–39]. Here, we ask how the two CI groups differ with respect to the time course and origin of the MMN under *difficult* phoneme perception conditions.

One possible outcome is that both groups activate similar regions in a comparable time interval, but strongly attenuated in comparison to controls, and more so for the poor performers. If this is the case, this argues for similar language processing mechanisms, but with greater or lesser success. If, however, the groups differ with respect to time course and neural generators, this is a further indication for different language processing mechanisms associated with good and poor performance [6]. Importantly, the use of the physically very same stimuli as in our earlier study allows us to draw conclusions about the brain signature to an identical stimulus appearing in either an easy or difficult context. If we observe profound differences between these situations, this is evidence for a context-specific adaptation. Both of these aspects depend on adaptation, learning and neural plasticity. The comparison of groups informs us about the neural correlates of good and poor performance. The contrast between the difficult and easy contexts provides information about context-specific adaptation.

**Fig 1:**
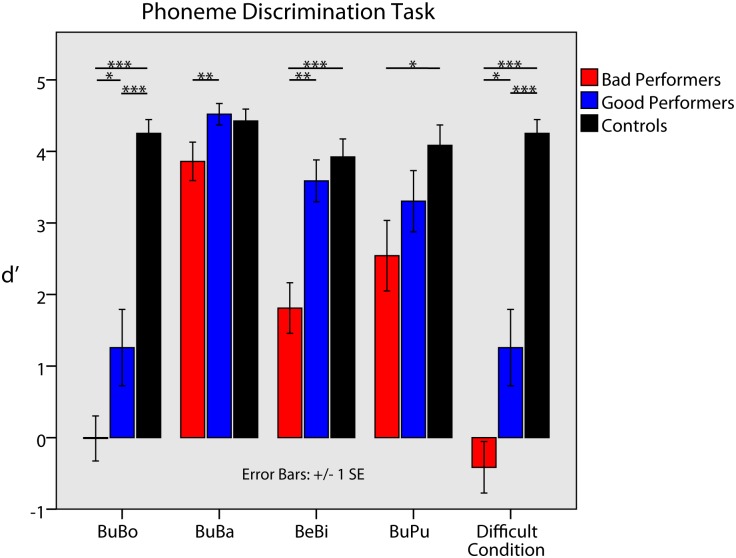
Performance in the phoneme discrimination test displayed for each subtest and all groups. While good performers achieved d’ scores that were close to the typical hearing control group (3 out of 4 subtests), poor performers showed significantly worse phoneme discrimination abilities than the good performers (3 out of 4 subtests). As intended, this changed when phoneme pairs were sorted for each participant’s most difficult condition. Here, good performers performed significantly worse than the control group and better than the poor performers, who only achieved a guessing level. T-values ranged from 1.7 to 15.21 with p-values from 0.05 (marked by an asterisk) to ≤ 0.01 (marked by two or more asterisks).

Please note that the current study reports on data that were collected at the same time as those reported in [6]. This implies that the participants, stimuli, pretests, methods of data processing and other aspects were the same in both reports, but the data (except for parts of Fig 1) are different. While [6] concentrated on the phoneme contrast that was most easy to discriminate, the current study focuses on results for the most difficult contrast. For the sake of completeness, the following method section includes all relevant information, and thus overlaps with [6].

## Materials and Methods

### Participants

#### CI users

To find pairs of prelingually deafened CI users that could be matched according to device experience and age at implantation, 64 patient files, stored in the archive of the Department of Phoniatrics and Pedaudiology of the Muenster University Hospital, were screened in detail. All CI users had to fulfill predefined logopedic and phoniatric criteria and have very good overall hearing abilities. Matched pairs differed only in their language performance: the group with high language performance was labeled “good performers”, the group with low performance “poor performers”. All potential candidates were intensively examined with respect to language perception and production by a speech therapist, who rated their articulation, syntax, morphology and semantics. An ordinal rating scale from 1 (high) to 6 (low) was used for all four sub-dimensions of language performance. Good performers had to score 2 or lower, while poor performers had to be rated 4 or higher. Thus, no overlap in language performance between good and poor performers was ensured. Information about the applied tests for all sub-dimensions of language performance is provided in S1 Table of the supplementary material, describing the characteristics that had to be fulfilled for each step of the ordinal rating in detail. To establish an index of global language performance, the criteria of all sub-dimensions were added up for each patient (range: 4–24 points). CI users that did not fulfil the strict criteria for group membership were excluded from the study and replaced by others. This way, 18 participants (nine pairs, eight female and ten male; aged 7–19 (mean = 12.9 years)) with equal educational background remained in the study. The mean global language performance was 6 points (4–8 points; std = 1.73) for good performers and 18.2 (16–20 points; std = 1.8) for poor performers. Device experienced was considered throughout and matched between groups.

All CI users had very good basic hearing abilities according to clinical standard testing. This was ensured by accepting only those that had at least 70% correct answers in the *Freiburg Monosyllable Word Test* (FMWT) [46]. This test assesses the ability to correctly repeat frequently used monosyllabic words in quiet, thus testing basic comprehension abilities for high-frequency words with only little involvement of compensational processes due to the semantic context. As opposed to producing spontaneous language, the accurate repetition of monosyllabic words is easier to manage. Therefore, although poor performers had trouble with articulation in *spontaneous* language, good results in the FMWT were achieved by all participants. In addition, we ensured very good hearing abilities by applying the MED-EL Teen-Ears test battery, a test-box designed to assess hearing in children and young adults after CI implantation [47]. Three tests were chosen. In test 1, CI users had to identify the number of syllables within spoken words (one, two or three syllables). In test 2, spoken sentences had to be identified from a set of written counterparts (“sentences in closed set”). Test 3 required the identification of key words of spoken sentences without the help of lip reading or presentation of their written counterpart (“sentences in open set”). In test 1 and 2, both groups had 100% correct answers; in test 3, good performers achieved 99% and poor performers 90% correct answers. S2 Table of the supplementary material shows the results of all language perception tests for each participant.

In all CI users, the best possible transmission of signals to the auditory cortex, especially in the speech relevant dynamic range between 35 and 65 dB, was ensured by optimizing the aided thresholds of each participant. For this, aided threshold were measured in the frequency range of the CI between 250 and 8000 Hz with third octave noise. The first sound was always presented at 60 dB HL and decreased in steps of 20 dB until the CI user reported not having heard any sound. Then, sound levels were increased in steps of 5 dB until the patient noticed the test stimulus again. The threshold value was validated by varying sound levels by variations of 5 dB steps below the initially identified threshold. Next, the volume of each frequency was increased until patients reported uncomfortable intensities or until 100dB HL were reached. By this procedure the physiological dynamic range was maximized while low level background was inhibited and uncomfortable signal peaks were prevented. Participants had received their last map change earliest two month before data assessment (max = 26 month, mean = 7.8 ± 5.1 month).

In short, using this intensive screening procedure, we put together two extreme groups with no overlap in language perception and language production (tested via global language performance as mentioned above: t_(8)_ = 14.4, p<0.001, n = 18), but with equal device experience and with very high hearing abilities.

All participating CI users had deafened prelingually and had no language perception with hearing aids. In both performance groups patients were provided with gestures at school, but with different success: while three of the good performers achieved low (two) to moderate (one) performance level, seven of the poor performers achieved either low (two) to moderate (four) performance with one of them showing even good abilities in the comprehension and use of gestures. All except one pair (pair 9) received their CI before their fifths birthday. Both patients of pair 9 had deafened prelingually and were offered CIs. However, their parents decided to try high-level hearing aids first, but later switched to CIs.

CI users were initially matched according to device experience and age at implantation rounded to the year. This resulted in no difference between groups concerning age at implantation (t_(8)_ = -0.55, p = 0.59; good performers: mean = 4.2, std = 4.84; poor performers: mean = 4.55, std = 3.32) and device experience (t_(8)_ = 1.35, p = 0.21; good performers: mean = 11.33, std = 4.06; poor performers: mean = 10.22, std = 3.34). Even when taking the exact age into account (years, months), no significant difference was found: device experience (t_(8)_ = 1.12, p = 0.27; good performers: mean = 4.14, std = 4.97; poor performers: mean = 4.5, std = 3.35); age at implantation (t_(8)_ = 0.59, p = 0.57; good performers: mean = 11.27, std = 4.16; poor performers: mean = 10.29, std = 3.36). However, if pair 9 is considered an outlier and excluded from analyses, age at implantation is significantly lower (t_(7)_ = 3.87, p = 0.006) in good performers (mean = 2.5 years, std = 0.81) than in poor performers (mean = 3.4 years, std = 1.11).

To learn more about the influence of age at implantation on global language performance, we calculated a Pearson’s Correlation test. As before, with pair 9 included, age at implantation had no influence in the current study group on global language performance (r = 0.125, p = 0.622). This changed when pair 9 was excluded; the correlation test then showed a trend towards significance (r = 0.49, p = 0.053), indicating a possible relationship between early CI implantation and favorable language development. Therefore, to control for age at implantation as a possible confounding variable, we included it as an additional variable (measured in years, months) in all relevant steps of data analysis. For further information on patient data and descriptive statistics, see [6].

Eleven CI users had bilateral CIs, while seven had unilateral implants. All participants were patients of the Department of Phoniatrics and Pedaudiology of the Muenster University Hospital, Germany. One pair had received their implant at the Hannover Medical School but was treated further in Muenster. Thus, across groups, all participants received the same type of rehabilitation treatment procedures.

All CI users were provided with CIs from Cochlear^®^ (Cochlear, Sydney, Australia), with nine using *Freedom* speech processors (seven poor performers, two good performers) and the other nine using an *Esprit 3G* (two poor performers, seven good performers). Note that good performers mainly used the *Esprit 3G* speech processor, the *precursor* of the *Freedom* processor, at the time of testing. In contrast, poor performers mainly used the *Freedom* processor as a follow-up to wearing the *Esprit 3G*. Physicians and parents had hoped for an additional gain in language performance due to a change in processor technology, which turned not out to be as successful as hoped. For further information on CI-related processing strategies and patient data, see Table 1.

**Table 1:** Subject Demographics of Both CI groups.

Pair	Group	Gender	Device Experience [years; month]	Age at Implantation [years; month]	Measured CI	CI-Speech Processor (measured)	Cause of Deafness	Age at Hearing Loss Onset	Progressive vs. Congenital	Ability to comprehend and use gesturing	Device Configuration	BERA Pre CI left/right [dB]	Averaged Pure Tone Audiogram Pre CI [dB]
1	GP	male	12;0	3;1	right	Esprit 3G ACE/1200	unknown	birth	congenital	low	unilateral	>110/>110	not available
	BP	male	13;2	4;4	right	Esprit 3G ACE/900	unknown	prelingual	progressive	low	unilateral	>100/100	not available
2	GP	male	10;10	2;1	right	Esprit 3G ACE/1200	connexin 26	prelingual (< 4 weeks)	unknown	no	unilateral	>95/>95	not available
	BP	male	9;3	4;2	left	Freedom ACE/900	unknown	prelingual (ca. 12 month)	progressive	no	bimodal	>100/90	>100/90
3	GP	male	7;7	3;3	right	Esprit 3G ACE/1200	unknown	prelingual	progressive	no	bilateral	85/95 (implanted at Hannover University Hospital)	not available
	BP	male	5;6	4;5	right	Freedom ACE/1800	unknown	birth	congenital	moderate	bilateral	>100/85	105/110
4	GP	female	12;0	3;6	left	Esprit 3G ACE/1200	cytomegaly	birth	congenital	low	unilateral	>100/>100	>100/>100
	BP	female	13;1	3;10	right	Esprit 3G ACE/1200	cytomegaly; peripartal hypoxia	prelingual	unknown	moderate	unilateral	>100/>100	>100/>100
5	GP	male	15;3	2;0	right	Esprit 3G SPEAK/250	unknown	prelingual	unknown	no	bimodal	not available (implanted at Hannover University Hospital)	>100/>100
	BP	male	14;9	3;3	right	Freedom SPEAK/250	unknown	birth	congenital	moderate	unilateral	>95/>95	>100/>100
6	GP	female	12	3;0	right	Freedom ACE/1200	unknown	pre/perilingual (18 month)	progressive	moderate	bilateral	70-80/60-80Later: >100/>100	>100/>100
	BP	male	9;2	4;0	right	Freedom ACE/1200	unknown	prelingual (ca. 12 month)	progressive	good	bilateral	>100/>100	>100/>100
7	GP	female	6;1	2;0	right	Esprit 3G ACE/1200	unknown	prelingual	unknown	no	bilateral	>100/>100	>100/>100
	BP	female	8;2	1;11	right	Freedom ACE/1200	unknown	birth	congenital	moderate	bimodal	95/>100	not available
8	GP	male	6;9	1;2	right	Esprit 3G ACE/1200	unknown	birth	congenital	no	bilateral	>100/>100	>90/>90
	BP	female	6;4	1;7	right	Freedom ACE/1200	unknown	birth	congenital	no	bilateral	>100/>100	>100/>100
9*	GP	female	with CI: 2;5, with hearing aid: 19	17;3	right	Freedom: ACE/900	unknown	prelingual	progressive	no	bimodal	>100/>100	90/>100
	BP	female	with CI: 2;3, with hearing aid: 13	13;0	right	Freedom: ACE/900	probably by ototoxic antibiotics	pre/perilingual (18 month)	progressive	low	bimodal	70/>100	80/>100
GP: Mean (SD)			11;3 (4;2)	4;2 (4;11)									
GP: Mean (SD) without pair 9			10;3 (3;2)	2;6 (0;9)									
BP: Mean (SD)			10;4 (3;4)	4;6 (3;4)									
BP: Mean (SD) without pair 9			9;11 (3;5)	3;5 (1;1)									

**Demographic Data and CI Specific Information:** Data were retrieved from each patient’s medical record. Pairs were matched according to device experience and age at implantation. All participants deafened prelingually. Those who had received only one CI and who wore a hearing aid contralaterally are denoted as “bimodal”. Please note that hearing aids did not improve performance in language perception tests and are no indicator for residual hearing.

The variance between CI users due to the number of CIs implanted (bilateral or unilateral) was controlled for by measuring only their first implant in bilaterally treated patients. Four of the unilaterally provided CI users used hearing aids on the contralateral side. Still, hearing aides had no beneficial effect on language perception. Taken together, in 16 CI users the right ear was assessed, while the left ear was assessed in two users. Although in most patients no hearing ability remained in the contralateral ear, all received an earplug during auditory testing.

#### Healthy controls

In addition to the two patient groups, phoneme discrimination abilities and the MMN were measured in an age-matched control group with typical hearing (four male, five female; 8 to 20 years, mean = 14 years). The same ear was stimulated in controls as in their matched CI partners; the other ear was closed with an earplug.

#### Ethics statement

All participants (or their parents in case children were younger than 18 years) provided their written informed consent to participate in this study. The study was approved by the Ethical Committee of the Deutsche Gesellschaft für Psychologie (DGPS) in conformance with the 2004 declarationof Helsinki.

#### General procedure

All CI users underwent a logopedic and phoniatric assessment by a speech therapist, which lasted 1.5 hours. Next, phoneme discrimination abilities were measured. EEG was recorded in a camera silens, a soundproof and electrically shielded chamber. Participants watched a silent movie of their choice during EEG registration. In sum, the complete procedure took five to six hours, including breaks.

### Data Assessment

#### Stimuli and stimulus presentation

Six German phonemes embedded in syllables were chosen as stimulus material: /bu/, /bo/, /ba/, /pu/, /be/, /bi/, all with tense vowels. Stimuli were spoken by a male adult recorded with Audacity 1.3.beta^®^ using a sample rate of 48,000 Hz and resolution of 16 bit. Cool Edit Pro 1.2a^®^ was used for further processing. Stimuli were cut to be as equal as possible in length, but to still sound like the intended syllable, varying from 420 to 451 ms in duration (mean = 435 ±14.5 ms). An onset of the sound file that sounded the most natural was chosen. Stimuli were carefully faded in to avoid sound artifacts. This resulted in a brief “silence” before language onset (depending on the stimulus, up to 50 ms, see Fig 1 in the supplementary material). To avoid clipping, stimuli were normalized to 95% of the maximum amplitude and equalized in average RMS values. Syllables were presented via two loudspeakers that were placed at an angle of ±20° azimuth approximately 1.5 m in front of the participant, who was seated in a comfortable chair.

Stimuli were delivered via Presentation 13.0^®^ (Neurobehavioral Systems, California, USA). Loudness was adjusted individually by presenting the syllable /bo/ at intensities using an audiometer (Medimate 622D from Otometrics, Taastrup, Denmark). A visual analog scale, which was especially designed for children and adolescents to rate categorical loudness, was used until the stimulus was reliably rated as “comfortably loud”, ensuring adequate und comparable stimulation levels across participants.

The most difficult-to-differentiate pair out of three (/bu/ vs. /ba/, /bu/ vs. /bo/, bu/ vs. /pu/; see Phoneme Discrimination Task below) was identified for each CI user and taken as the stimulus pair for EEG registration. Stimuli had an average length of 436 ms and an inter-stimulus interval of 900 ms (with a jittering of ± 200 ms). In 17 CI users, /bu/ vs. /bo/ was used, in one user, /bu/ vs. /pu/ was used. Healthy controls were always stimulated with the phonetically most similar and therefore most difficult to discriminate pair, /bu/ vs. /bo/ [48]. Please note that /bu/ was presented to all participants in this design. Moreover, it was used as both standard and deviant, the order balanced across participants.

To identify the point in time at which those 26 participants, who were presented with /bu/-/bo/, were able to reliably distinguish the target stimulus from the distractor, we performed a gating task [49]. 20 participants (students of the University of Muenster, 10 female, aged 20–30, Ø24 years) were presented with fragments of increasing duration of the sound files /bu/ and /bo/, starting at 70 ms and up to 350 ms, with increments of 20 ms. Participants had to identify the corresponding syllable in a forced-choice task by pressing a button. Hits for all fragments of the stimulus /bu/ were assessed. The point of identification was determined for each subject as the fragment at which the identification was correct and not changed on subsequent fragments. These identification points were then averaged across participants and served as an indicator of the time point within the stimuli at which the MMN could be triggered. Data analyses revealed that, on average, 250 ms of /bu/ was sufficient for successful discrimination from /bo/ (std = 91 ms). Given that the MMN is triggered by differences between standard and deviant, we used this duration as an index for the onset of the MMN. Therefore, MMN latencies were reduced by 250 ms. In the results section, we present both latencies–corrected and uncorrected. In the discussion, we only consider corrected latency (lc) times. With only one out of 27 participants having received /bu/-/pu/, a corresponding procedure in the gating task was abandoned.

#### Behavioral data and ratings

*1*. *Phoneme Discrimination Task*: Phoneme discrimination abilities were assessed by presenting four stimulus pairs varying in phonological similarity: /bu/ vs. /bo/, /bu/ vs. /ba/, /bu/ vs. /pu/ and /be/ vs. /bi/, with /bu/ appearing in three out of four pairs. This was done to identify the most difficult-to-discriminate stimulus pair in each individual CI user, which was then used in the MMN registration. Note that /bu/ served as the target stimulus for all participants, as standard and deviant. /be/ vs. /bi/ was used to extend phonological discrimination beyond the vowel /u/ (but was never selected as the most difficult pair).

The order of the four subtests was randomized across CI users. Participants had to differentiate stimulus pairs in a forced-choice design by deciding which stimulus had just been presented via mouse click. Each test consisted of 60 repetitions, with equal probabilities of occurrence for both stimuli. Hits, misses, correct rejections and false alarms were used to calculate the sensitivity index d’.

*2*. *Subjective Satisfaction Rating*: To identify personal satisfaction with language perception, CI users self-rated twelve items concerning their ability to function in daily communication situations; for example: “*How often do you talk to somebody on the phone you don’t know*?*”*. Items were ordinally scaled (5-point rating scale) with the levels: “never”, “rarely ever”, “sometimes”, “often” and “always”. Together, scores ranged between 12 and 60 points. Questions were adapted from the *Manchester Teen Questionnaire*, which is part of the *TeenEars Testbox* [47]; see S3 Table of the supplementary material.

#### Eeg data recording

An odd-ball paradigm with a ratio of 85:15 (standard:deviant, in percent) was used to evoke the mismatch negativity, with two runs, resulting in 803 standards and 141 deviants per run. Each run lasted approximately 20 minutes.

Participants watched a silent movie of their own choice. Brain responses were measured with a 32-channel EEG cap (model “Easycap BrainCap-MR 3–0 32Ch) using electrodes that were located according to the international 10–20 system. FCz was defined as the reference channel, eye blinks were recorded with an EOG electrode that was placed at a central position below the right eye. Electrode impedances were always kept below five kΩ. Electrodes located above the CI (on average 2.8 electrodes per subject) were left out of the analyses. This procedure was shown not to influence source localization in CI users [40,50]. Passing an amplifier (Brain Amp MR, 32 channels), data were filtered online with 0.1–250 Hz and recorded with 500 Hz using Brain Vision Recorder^®^. Additionally, participants’ individual electrode positions were digitized using Polhemus Fastrack^®^ 3D to account for inter-individual differences.

### Data Processing and Analyses

#### 1. Behavioral data and ratings

*Phoneme Discrimination Task*: In addition to the sensitivity index d’ that was individually calculated for each stimulus pair, a discriminant analysis was computed to investigate whether individual performance in the difficult condition of the phoneme discrimination task could correctly predict group membership. A repeated measures ANOVA was calculated to identify differences between groups. Two-tailed paired t-tests were used to investigate effects more in detail [51].

#### Eeg data analysis

EEG data were imported into BESA 5.3^®^ and epoched from -200 to 600 ms, to analyze the Mismatch Negativity. Each CI user’s electrode position was normalized to account for differences in head forms, and dead channels were interpolated. To avoid excessive loss of trials due to the poor quality often found in patient data, eye-blink distorted trials were not simply rejected, but corrected using the adaptive artifact correction method provided by BESA^®^ [52]. This method models not only the artifact that has to be excluded (defined here by manually marking a typical eye blink, followed by automatic identification of other blinks, which are then averaged to obtain the typical waveform and topography of the artifact), but also models the brain activity to be retained. This way, brain activity and artifact are disentangled and brain waves, especially in frontal EEG channels, are not distorted. Blink-free EEG data were baseline-corrected from -150 to 0 ms and filtered from 0.1 to 25 Hz. All other artifacts (as e.g. due to movement) were removed using the artifact scan tool implemented to BESA^®^. Standards immediately preceded by a deviant were excluded from analyses. In good performers, 72% of all standards and 90% of deviants remained for analyses; in poor performers, this was 67/77%, in controls 70/87%. Next, the reference was set from FCz to average reference, electrode positions were transformed into standard positions and data were exported via MATLAB 2009a^®^ to Emegs2.5^®^ [53].

In Emegs, cortical sources of the event-related fields were estimated using the L2-Minimum-Norm-Estimates (L2-MNE) method for standards and deviants separately [54]. The L2-MME is an inverse modeling technique applied to reconstruct the topography of the primary current underlying the electric field distribution. It allows the estimation of distributed neural-network activity without a priori assumptions on the location and/or number of current sources [55]. In addition, of all possible generator sources, only those exclusively determined by measured electric fields are considered. Calculation of the L2-Minimum-Norm-Estimates was based on a spherical four-shell isotropic volume conductor head model, with three (radial, azimuthal, and polar direction) × 127 evenly and spherically distributed dipoles as source model. A source shell radius of 6 cm was chosen as trade-off between depth sensitivity and spatial resolution (Tikhonov regularization parameter k = 0.1). This distributed source reconstruction in EEG does not precisely localize cortical generators, but allows an approximation of cortical generators and a corresponding assignment to larger cortical structures.

In the control group, the MMN was established by calculating a point-wise repeated-measures ANOVA computed for each dipole and time point using the within-factor “stimulus” (standard vs. deviant). The control group provided a reference for the time course and the localization of the MMN in participants with typical hearing.

To identify the MMN in CI users and to find differences between good and poor performers regarding the MMN, another point-wise ANOVA with the within-factor “stimulus” and the between-factor “group” was performed (ANOVA 1). The main effect “stimulus” served as an indicator for the MMN and the interaction “stimulus x group” displayed significant group differences. To investigate the interaction of group and stimulus in more detail, an additional point-wise ANOVA with the between-factor “group” was calculated, using the *difference wave forms* derived by subtracting averaged standards from deviants in each individual CI-user (ANOVA 2). Thus, this ANOVA was performed on the MMN response.

Each point-wise ANOVA resulted in a spatiotemporal distribution of statistical values for each dipole and time-point. Clusters displaying significant F-values (p<0.05) over at least ten consecutive data points and five adjacent diploes were chosen for further statistical analysis. In case of unilateral activation, significant clusters were mirrored to homologous regions of the other hemisphere to test for hemispheric differences and their interactions. For this, data of the mirrored clusters were averaged cluster-wise and exported to SPSS^®^19. Another repeated-measures ANOVA was calculated with the within-factor “stimulus” (ANOVA 1)/between-factor “group” (ANOVA 2) and the within-factor “hemisphere”. If necessary, post-hoc comparisons were performed with paired t-tests. Additionally, Pearson’s correlation tests were performed for each cluster, with phoneme discrimination (for all participants), individual satisfaction with his or her language perception and age at implantation (for CI users) as variables.

Differences in MMN strength between controls with typical hearing and CI users were roughly tested by calculating a repeated-measures ANOVA with the between-factor “group” and the within-factor “stimulus”, using the average amplitude of both group’s MMN (averaged across different clusters, if necessary).

#### Artifacts evoked by the implant

Using the identical stimulus as deviant *and* standard, the resulting MMN as the subtraction of standard and deviant is argued to be free of pure stimulus differences [56]. This also has the advantage that deviants and standards produce an identical CI artifact, which can then be eliminated by subtracting standards from deviants, as has already successfully been done [6,44].

To acknowledge the possible influence of the CI artifact on the MMN time course and localization, the neural signature of the artifact is displayed in time (Fig 3A) and space (Fig 5C). Artifact localization was done in standards and deviants.

All variables were tested for normality via the Kolmogorov Smirnov test. P-values for results were Greenhouse-Geisser corrected for non-sphericity (if necessary). In effects that were not derived from hypotheses, p-values of multiple paired t-tests/multiple correlation tests were bonferroni-corrected within each cluster [57]. To avoid excessive information, bonferroni correction was mentioned only in case the corrected t-test lost significance (by reporting the adjusted critical p-value (p_crit_)). All performed t-tests were two-tailed [51].

## Results

### Behavioral Data and Ratings

#### Phoneme discrimination task

Statistical analyses showed that control participants had excellent phoneme discrimination abilities across all phoneme pairs and good performers a similar performance level. Poor performers did significantly worse than good performers (please note that these results were originally published in [6], therefore a detailed statistical report is not given here). Sorted by each person’s most difficult-to-differentiate subtest, the three groups showed a different performance: while control participants performed on a very high level (d’ = 4.25, which equals about 99% correct answers), good performers showed moderate performance (d’ = 1.25, 68% correct answers) and poor performers performed on guessing level (d’ = -0.41, equaling about 47% correct answers). Therefore, there was a significant decrease in the ability to discriminate the most difficult phoneme pair from the control group to the good performers (t_(8)_ = 5.2, p = 0.001) and from good to poor performers (t_(8)_ = 2.67, p = 0.03). Statistical differences between groups and across all subtests are displayed in Fig 1. These results clearly demonstrate the validity of phoneme discrimination tests to distinguish good and poor performers at a group level.

#### Discriminant analysis

The predictive value of phoneme discrimination abilities was tested on an individual basis via a discriminant analysis. Of the two variables (d’ of phoneme discrimination in the difficult condition, and age at implantation), only the first entered the model (entry criterion: F = 3.84, remove criterion: F = 2.71, F_(1,16)_ = 74.17, p<0.001), with the result that every single CI user was correctly sorted into his or her performance group. On a single-case level, this impressively confirms the power to predict a group membership that was established by means of a large test battery with only one variable indexing phonological awareness. The result did not change when pair 9 was excluded (F_(1,14)_ = 56.75, p<0.001).

### EEG Data

#### Sensor space

**Fig 2:**
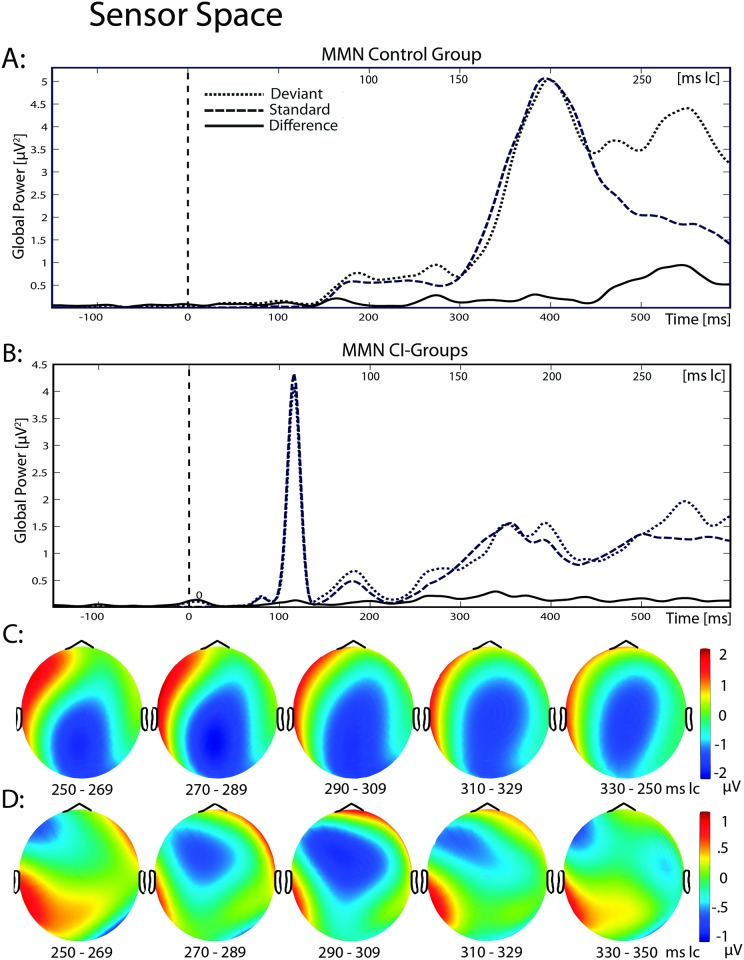
MMN in sensor space. Global power plot for standards, deviants and their difference waveform (deviant minus standard) of the control group (A) and averaged for both CI groups (B). The difference waveform of the controls shows a distinct MMN, with a peak at approximately 280 ms, which was far less prominent in CI users (B). Here, the CI artifact is clearly visible in standards and deviants from 100 to 130 ms, but not in their difference waveform. (C/D): topographic map of the MMN across time with a central negativity displaying the MMN in the control group (C) and–though less pronounced–in CI users (D). Data were assessed with average reference.

To be able to compare the MMN to results to previous ERP studies, standards, deviants and their difference waveforms are displayed for all three groups in sensor space, in terms of a global power plot for standards, deviants and the difference waveforms. This is done for the control group (Fig 2A) and the averaged CI groups (Fig 2B), and as topographic maps across time for the difference waveform of the control (Fig 2C) and the averaged CI groups (Fig 2D).

#### Source space

**Fig 3:**
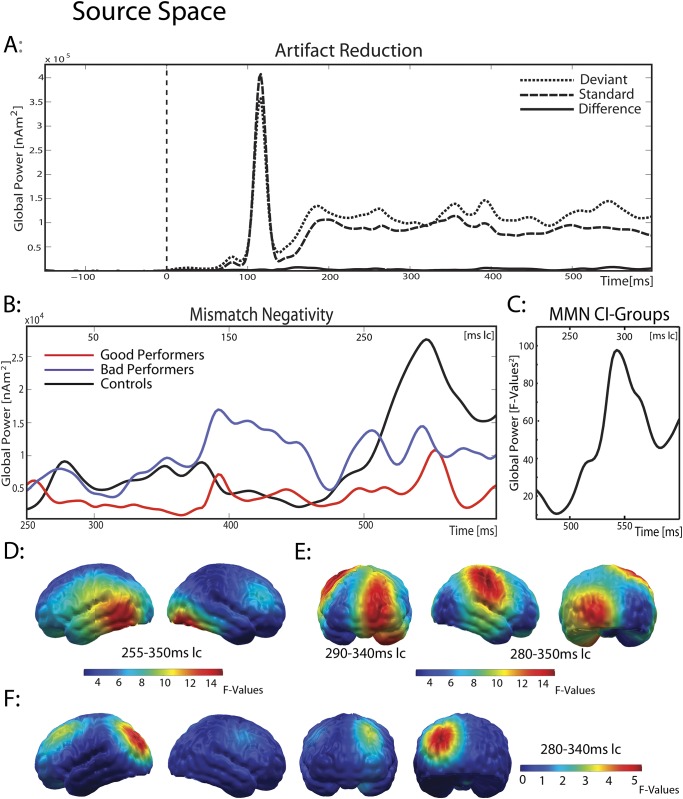
MMN in source space. (A) global power plot for minimum norm estimates of the CI group, separately displayed for deviants, standards and their difference (MMN). The artifact is clearly visible in standards and deviants from 100 to 130 ms, but–as in sensor space–not in their difference waveform. (B) Global power plot of the difference waveform for all three groups and (C) averaged across both CI groups, with a focus on the MMN time window. (D/E) Topographic map of the MMN in the control group and (D) averaged for both CI groups (E). While the control group showed a bilateral MMN in temporal and supramarginal regions, CI users engaged not only auditory, but also frontal and occipital regions during MMN. (F) Poor performers showed (a trend) for significantly higher activity in the left occipital cortex during MMN.

Fig 3A separately displays the global power of the L2-MNE for deviants, standards and their difference waveform, averaged for all CI users. While standards and deviants both clearly show the impact of the artifact caused by the implant, their difference waveform is free of this artifact. The artifact was identical in deviants and standards, and appeared between 100 to 130 ms with a maximum at 114 ms. As a consequence, it completely disappeared after calculating the deviant-standard difference. Fig 5C shows the artifact localization in the 16 CI users who wore their implant on the right side. As expected, it was correctly located in the right temporal lobe and, as demonstrated below, did not confound the MMN results in the spatial or in the time domain.

*MMN of the control group*: Fig 3B separately displays the global power of the MMN (deviant minus standard) for all three groups. Inspection of the amplitude of the MMN showed that the control group displays a stronger MMN than both patient groups. The time course of the MMN in the control participants (black line) revealed a MMN from 470 to 600 ms (220–350 ms latency corrected (lc)), with a peak at around 550 ms (300 ms lc). Projecting the L2-MNE source localization for this time interval onto a cortical surface, a bilateral activation in temporal regions was found from 505 to 600 ms (255–350 ms lc), consisting of 19 dipoles each (main effect “stimulus”: F_(1,8)_ = 21.04; p<0.01), with a trend for a hemispheric dominance of the left hemisphere (“stimulus x hemisphere”: F_(1,8)_ = 3.95; p = 0.08, t_(8)_ = 1.99, p = 0.08, see Fig 3D). The sources of the left and right hemisphere did not correlate with phoneme discrimination abilities in the difficult condition (left: r = -0.24, p = 0.53; right: r = -0.11, p = 0.78, N = 9).

*MMN of the CI users*: Given the latency and the regions of interest (ROI) predefined by the literature and by the MMN of the control group, the MMN of the CI users was expected in fronto-temporal regions, starting not earlier than 470 ms (220 ms lc). Fig 3C displays the Global Power plot of the main-effect “stimulus” from ANOVA 1, showing the MMN in CI users from 500 to 600ms (250–350ms lc), with a clear peak at about 550 ms (300ms lc). Fig 3E shows the result of ANOVA 1 averaged for both patient groups and projected on a cortical surface. The MMN was located within three different regions: the left prefrontal cortex, the right temporal cortex with strong involvement of the supramarginal gyrus, and the left occipital cortex.

Follow-up analyses revealed highly significant effects of the MMN in the frontal cluster from 540 ms to 590 ms (290–340 ms lc; cluster 1, main effect “stimulus”: F_(1,16)_ = 19.64; p<0.001; 29 dipoles; Fig 3E left) with no hemispheric dominance (“stimulus x hemisphere”: F_(1,16)_ = 2.78; p = 0.12). The same was found for the right temporal and supramarginal gyrus from 530 to 600 ms (280–350ms lc; cluster 2, main effect “stimulus”: F_(1,16)_ = 14.56; p = 0.002; “stimulus x hemisphere”: F_(1,16)_ = 0.76; p = 0.4; 13 dipoles; Fig 3E middle). In contrast, the MMN component located in the occipital cluster (cluster 3, main effect “stimulus”: F_(1,16)_ = 11.45; p = 0.004; 8 dipoles; Fig 3E right) was dominant to the left hemisphere (“stimulus x hemisphere”: F_(1,16)_ = 10.7; p = 0.005, standard vs. deviant in the left hemisphere: t_(17)_ = 2.97, p = 0.009), but not for the right hemisphere (p>0.5). In every cluster, deviants showed stronger activity than standards (deviants ranging on average from 464 to 584 nAm (std: 167–190), standards from 303 to 413 nAm (std: 104–161).

*MMN–CI users vs*. *control group*: To estimate group-specific differences in stimulus processing between CI users and the control group, a repeated-measures ANOVA with the between-factor “group” (controls vs. CI users) and the within-factor “stimulus” (standards vs. deviants) was computed, revealing a significant main effect for the factor “stimulus”, with stronger activity in deviants than in standards across groups (F_(1,25)_ = 42.7; p<0.001; deviants = 663 nAm; standards = 437 nAm). There was no difference in stimulus processing between CI users and controls with typical hearing (stimulus x group: F_(1,25)_ = 1.37; p = 0.25). Remember that this was just a crude estimation of MMN amplitudes in the groups, with MMN amplitude averaged across all group-specific MMN-related clusters.

**Fig 4:**
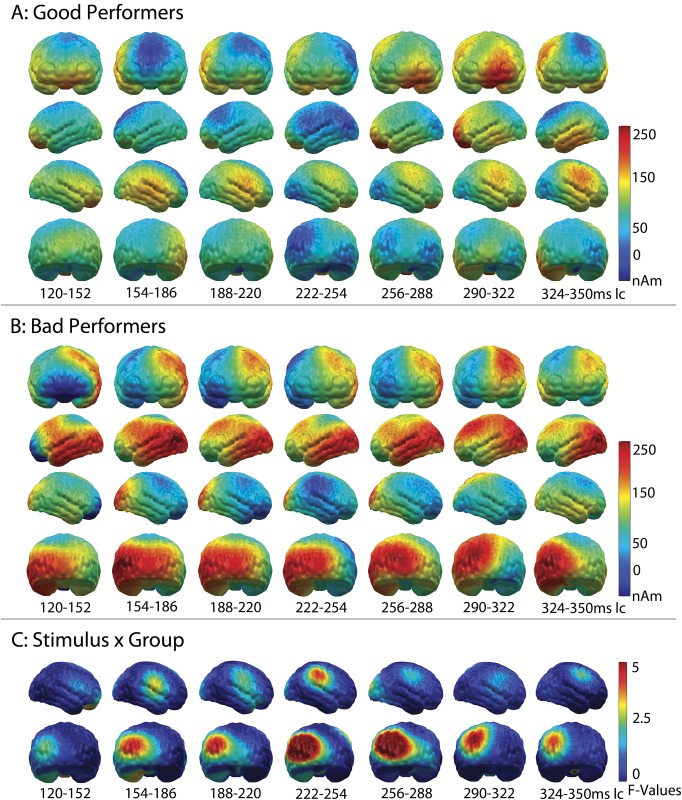
Source localization for the difference waveforms of the good (A) and poor (B) performers across time. Visual impression that both groups differ regarding the activation of the right temporal and right supramarginal cortex (GP>BP) before the MMN begins and in the left occipital cortex (BP>GP) during and before the MMN is underlined by statistical analyses (C).

Fig 4A and 4B separately show the L2-MNE of the MMN difference wave-forms projected on a cortical surface for the two patient groups. Visual inspection suggests that the prefrontal component of the MMN is equally strong in both groups, while the right temporal component seems to be enhanced in good performers. In contrast, the left occipital component seems more prominent in poor performers. Interestingly, these group differences seem not to be restricted to the time range of the MMN, but also include those preceding the MMN.

*MMN—good vs*. *poor performers*: To investigate these differences, a second point-wise repeated-measures ANOVA was calculated on the difference waveforms (deviants minus standards, ANOVA 2, for results, see Fig 4C), with the between-factor “group”. Hemispheric dominance was investigated by mirroring significant clusters to the other hemisphere. During the MMN time window, no significant group differences emerged in the main regions of interest–the prefrontal or auditory cortex. A significant interaction was only observed for the left occipital component of the MMN (group x hemisphere: F_(1,16)_ = 8.04; p = 0.01, “cluster 4”, 7 dipoles, see Fig 3F). Post-hoc analyses revealed a trend towards significance for stronger activity in the poor performers than in the good performers, from 530 to 590 ms (280–340 ms lc) in the left occipital regions (t_(8)_ = 2.12, p = 0.07), which was not found in the right hemisphere (t_(8)_ = 0.57, p = 0.59).

**Fig 5:**
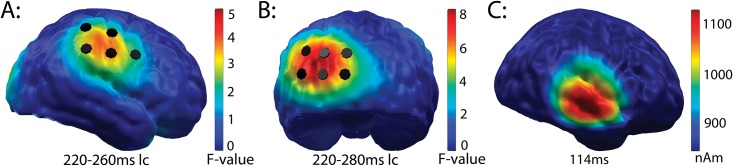
Source localization for the significant group differences found before the MMN began. While good performers showed significantly higher activation from 220 to 260 ms in the right marginal cortex (A), poor performers activated their left occipital cortex more strongly from 220 to 280 ms. This allows the conclusion that both groups engaged different neural patterns for the processing of difficult-to-differentiate phoneme pairs. (C) Correct source localization of the averaged CI artifact in the 16 participants who wore their CI on the right side.

*Differences preceding the MMN- good vs*. *poor performers*: Besides this difference in the MMN window, the point-wise ANOVA identified two significant group effects, both directly preceding the MMN. In the area of the right supramarginal gyrus, this difference was found from 470 to 510 ms (220–260 ms lc) in five dipoles (see Fig 5A); in the left occipital cortex, it was seen from 470 to 540 ms (220–280 ms lc) in six dipoles (see Fig 5B). Adjacent dipoles were combined and labeled “cluster 5” (right supramarginal gyrus) and “cluster 6” (left occipital cortex).

To further investigate hemispheric dominance, clusters were mirrored to the other hemisphere. For cluster 5, a repeated-measures ANOVA showed a significant interaction for group x hemisphere (F_(1,16)_ = 8.3; p = 0.01). Post-hoc tests revealed significantly stronger activity in the right supramarginal gyrus in good performers than in poor performers (t_(8)_ = 2.75, p = 0.02), while no difference was found in the left homologous region (t_(8)_ = 1.03, p = 0.33). In the left occipital cortex, a repeated-measures ANOVA showed a trend toward significant interaction (group x hemisphere; F_(1,16)_ = 3.64; p = 0.07). Post-hoc t-tests revealed that activation in the left hemisphere was significantly larger in poor than in good performers (t_(8)_ = 2.87, p = 0.02). This was not found in the right hemisphere (t_(8)_ = 1.17, p = 0.28).

#### Correlation analyses

**Fig 6:**
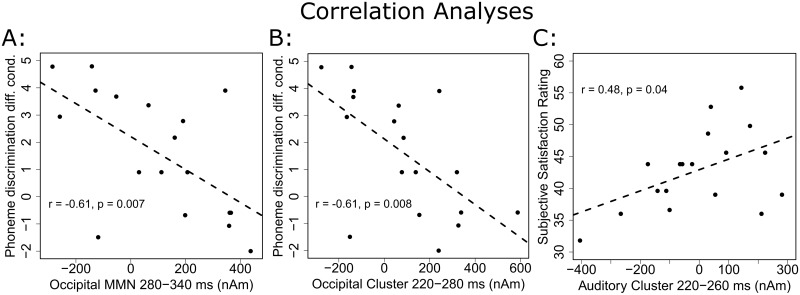
Correlation analyses. Activity in the occipital cluster during (A) and before (B) the MMN correlated negatively with phoneme discrimination ability in the difficult condition. The stronger CI users activated their left occipital cortex, the poorer their performance was in the phoneme discrimination test. In contrast to that, there was a trend for higher activity in the right supramarginal cluster being associated with more satisfaction regarding the CI user’s subjectively perceived language intelligibility.

To explore the relations between brain activity and behavioral performance, we correlated regional activities with behavioral responses. For these group-differentiating clusters, activity of the left occipital cortex during the MMN (cluster 4) was related to the behavioral performance for phoneme discrimination in the difficult condition (r = -0.61, p = 0.008, N = 18), which shows that the weaker phoneme discrimination was established in CI users, the stronger the left occipital cortex was activated during the MMN (Fig 6A).

Interestingly, the same correlation was found before MMN onset: again, the left occipital cortex (cluster 6) highly correlated with phoneme discrimination abilities in the difficult condition (r = -0.61, p = 0.007, N = 18, Fig 6B). In the right supramarginal gyrus (cluster 5), a trend for a significant correlation with individual satisfaction with one’s own language perception (r = 0.48, p = 0.04, Bonferroni correction: p_crit_ = α/3 = 0.016, N = 18) indicated that the more strongly the right supramarginal gyrus was activated before MMN onset, the more satisfied a patient was with his or her language perception (Fig 6C).

Brain activity did not correlate with age at implantation during or before the MMN (all r < 0.18, all p > 0.46, N = 18). The CI user who was stimulated with /bu/ vs. /pu/ instead of /bu/ vs. /bo/ was tested for being an outlier within each cluster (defined by two standard deviations from mean activity). Except for the left occipital activation that preceded the MMN (cluster 6), there was no difference compared to other CI users. Repetition of all tests involving cluster 6 showed no changes in results when the outlier was removed.

## Discussion

Our goal was to investigate phoneme processing in a difficult context in CI users that had developed either good or poor language understanding skills after cochlear implantation in early childhood. Due to its core function in language processing, we used phoneme discrimination and measured the mismatch negativity as its neurophysiological correlate. The cortical origin of the electrophysiological response was determined with distributed source estimates. The behavioral results confirmed the value of phoneme discrimination for the differentiation of persons with either high or low performance in general language functions. The neurophysiological analyses demonstrated that the MMN in CI users is similar in latency and overall amplitude to the MMN of matched controls. However, while the MMN in controls appeared bilaterally in fronto-temporal regions from 255 to 350 ms, CI users showed their MMN from 280 to 350 ms in frontotemporal and occipital regions. This finding is in line with brain-imaging studies that reported a pronounced occipital activity in CI users under purely auditory conditions. These findings were taken as evidence for cross-modal perception, a consequence of lip reading as a strategy for language comprehension [58]. Differences in language processing between good and poor performers were only marginal in the MMN window, but significant before typical time ranges of the MMN. *During* the MMN, there was a trend towards a stronger activation of the left occipital cortex in the poor performers that was most significant immediately *before* the onset of the MMN (220–290 ms). Additionally, the good performers recruited the right supramarginal gyrus, which has been reported to be involved in the analysis of phonemes [59,60].

Contrasting these results with our earlier study on phoneme discrimination in an easy context suggests that, even after successful language development, CI users use different language processing strategies that are strongly context dependent. Importantly, we provide evidence for this dissociation in response to the very same stimuli, presented in different language contexts. Taken together, our results emphasize the role of neural plasticity and adaptation in language development after CI implantation. In the following, we discuss each of these aspects as well as their implication for clinicians.

### MMN Amplitude, Localization and Latency

Distributed source localization revealed that the MMN in CI users was localized in fronto-temporal and occipital regions. Hemispheric dominance was only found for the left occipital cluster. Except for this cluster, the cortical origin of the MMN fits with the literature reporting bilateral fronto-temporal activations [29,31]. It also fits with our earlier results for the same deviant, when it was easy to discriminate from the standard (/bu/ vs. /ba/). In that study, the MMN also appeared in fronto-temporal regions, but hemispheric dominance was far more prominent [6]. This may indicate that recruitment of both hemispheres is needed when language perception is difficult. The activation over temporal regions, only observed in the current study, involved the supramarginal gyrus, a region known to be highly involved in phoneme discrimination [59–61]. Another difference concerns the amplitude of the MMN which was strongly attenuated relative to study 1. We believe that this reduction is due to the difficulty to distinguish the presented phonemes, given that the MMN is reduced when auditory items are hard to differentiate (e.g., [62]), an effect that is especially strong in CI users [35].

With a latency of 220 to 350 ms, the MMN of our control participants with typical hearing appeared late and was rather broad. This was expected for reasons detailed in our earlier study [6]: for example, subtle differences between standards and deviants are reflected in delayed MMN latencies (e.g., [62]). Also, children and young adults aged 7 to 19 years were tested, and it is known from developmental studies that ERP latencies decrease over the course of maturation [63,64]. Given that the age range of our children was rather large, we assume that this led to the long-lasting MMN. Moreover, phonemes trigger a rather broad MMN [33,65,66], in children as well as in adults [67].

In 17 out of 18 CI users, /bu/ vs. /bo/ was identified as the most difficult to differentiate phoneme pair. Only in one user, this was /bu/ vs. /pu/. Based on the gating task that was performed by 20 university students with typical hearing, the time window in which the MMN was expected to appear, was defined. Still, the gating task was performed only for /bu/ vs. /bo/, neglecting the one participants who had received /bu/ vs. /pu/ instead of /bu/ vs. /bo/. It can be assumed, that a gating task performed on /bu/ vs. /pu/ would have resulted in an earlier point of identification, due to the different consonants at the beginning of both syllables. Therefore, also the MMN should have been triggered earlier in this participant. This possibly added more variance to the data. Still, further statistical testing did not reveal an influence of stimulation on the effects found in the EEG. As a consequence, the one participant who had received /bu/ vs. /pu/ instead of /bu/ vs. /bo/ did not change the results of the current study.

### Language Processing in Good and Poor Performers

We found no significant difference between good and poor performers regarding the overall amplitude of the MMN. This was surprising, because it did not reflect the behavioral evidence for phoneme discrimination, which was much better in good performers. Given that the MMN–although amplitude reduced (cf. [6])–was comparable in both patient groups, it seems that other group specific information processing strategies were employed when phoneme discrimination is difficult. These might be relevant to understand why some patients developed well and others did not.

For a MMN to be generated, a thorough evaluation of the sensory input is necessary beforehand [68]. If standards and deviants show only subtle differences, this early sensory analysis is likely to be intensified. This may explain why we found differences between groups prior to the MMN time range that was defined on the basis of a gating task. Note also, as briefly described in the introduction, that deviant detection correlates with earlier neurophysiological components than the MMN and its typical time range [34]. We use the term MMN here in reference to the large body of existing literature, but we regard it an open issue if this terminology with relatively fixed time intervals will hold for the future. We observed an enhanced activation in the good performers of the right supramarginal gyrus from 220 to 260 ms, directly preceding the time window of the MMN. Studies on word processing showed that the supramarginal gyrus is involved in phonological processing, especially in phoneme discrimination and phoneme categorization [59–61]. We thus suggest that this region is activated in good performers in order to enhance phonological processing and to correctly classify phonemes. A similar right-lateralization of the supramarginal region as observed here was reported in postlingually deafened CI users [69]. [70] showed increased activation of the right supramarginal gyrus in phonological processing that was accompanied by decreased involvement of the left supramarginal gyrus. Moreover, they observed decreased activation of the right supramarginal gyrus during processing of environmental sounds. This was interpreted as a compensation strategy that valued the processing of language stimuli as more important than that of environmental sounds. Naturally, this adaptive process resulted in worse language perception after CI activation than in those postlingually deafened CI users who solely relied on their left supramarginal gyrus to perceive language. In the current study with *pre*lingually deafened CI users, who received their implant before their fifths birthday, activation of the right supramarginal gyrus during phonological processing was associated with *better* language development. This indicates that the success of alterations in the functionality of brain areas in CI users highly depends on prior experience and task affiliation of the affected regions.

In contrast to the involvement of the supramarginal gyrus in good performers, poor performers displayed enhanced left occipital activity preceding the MMN. We assume that both groups recruit the visual cortex during the MMN for the interpretation of spoken verbal stimuli that are difficult to differentiate. Activity over visual regions under purely auditory conditions is a phenomenon repeatedly reported in postlingually deafened CI users using methods such as PET [71]. Based on these studies, we interpret the stronger activity in the left occipital lobe in poor performers during (as a trend) and before the MMN as the consequence of compensative strategies to gain additional information by e.g. lip reading [72] or other visual input as e.g. sign language. This highly trained strategy leads to cross-modal activity [73], even in the absence of visual input [71,74]. As can be seen in Table 1 most of our patients were trained with more or less success in using non-verbal means to communicate.

But how did these group-specific differences in language processing develop? Based on the literature, we see three possibilities: if the cross-modal activation constitutes a compensatory mechanism that was established to cope with processing difficulties in poor performers, it could be concluded that the two patient groups were not as well matched as intended, and differed in functions that we could not assess, as e.g neuronal survival in the auditory nerve or brainstem nuclei. Also maladaptive cortical plasticity as e.g. an occupation of the auditory cortex by visual functions (as described by [75]) could have prevented good development of phoneme representation in the auditory cortex as soon as hearing was rebuild. As such, it is possible that poor performers had problems after CI activation to process auditory cues properly and—as a consequence- they may have been forced to rely more strongly on lip reading, gesturing or German sign language. Until now, this is merely an assumption, as we have no data on how both groups might differ. All patients were extensively tested before surgery to ensure their suitability for the use of CIs (e.g., logopedic and phoniatric assessment, audiometry, anatomy of the outer, middle, inner ear, and auditory nerve, etc.). They underwent almost identical rehabilitative treatment after implantation in our clinic, though it cannot be ruled out, that poor performers received distinct training in comprehension and use of gestures at school. This would be supported by the fact that five out of six CI users with moderate to good competence in the comprehension and use of gestures were poor performers, while good performers showed twice as often no or only low performance (8:4). Still, this communication mode is only used in language therapy to facilitate language comprehension. There was no patient who was knowledgeable in German sign language.

Another possibility is that cross-modal activation was initially established to use visual cues to support auditory language processing, which resulted in a learned-non-use phenomenon in the long run (for a review, see: [76]). This phenomenon is well known in hemi-paretic stroke patients, for whom compensatory processes generally favor the easiest way to reach respective goals. In motor-impaired patients, this is evidenced by the preferred use of the unaffected arm, due to its temporary functional superiority, even if it is the non-dominant one. Over time, this leads to a complete lack of use of the affected arm, even after cortical recovery. In prelingually deafened CI users, the same phenomenon could have occurred in the poor performers. Before CI surgery, the visual channel was the main source of information for language. Interestingly, even prelinguistic infants integrate auditory and visual language information and thus make use of cross-modal perception to acquire language [77]. In CI users, due to years of intense practice, this strategy is highly trained and very effective. After CI activation, signals from the auditory channel were unfamiliar and their interpretation very complicated. Therefore, patients who developed into poor performers might have persisted in focusing on lip reading or other visual co-information as e.g. gesturing, thus further practicing the cross-modal interpretation of language signals, while patients who developed well concentrated more on phonological analyses.

Third, poor performers could have simply been less used to the new processing strategy following the last adjustment of their CIs aided thresholds. The average amount of time needed to habituate to changes in the aided thresholds is around two to three weeks. Therefore, less time could have resulted in lower performance in e.g. the phoneme discrimination tests. Still, this would not explain differences in language performance. Also, all CI users had received their last adjustment at least 2 *month* (average 7.8) before taking part in our study, as a consequence, we assume, that all CI users were fully used to their current map settings.

Apart from these arguments, there are two other factors in which our groups differ that might explain the results. One concerns technological equipment. Poor performers more often used a more modern speech processor technology (Freedom, 7:2) than good performers (Esprit 3G, 2:7). But as mentioned in the methods section, poor performers received the Freedom processor as a follow up to the Esprit 3G to further improve language perception, while the good performers showed satisfying results with the older processor technology and therefore had no need for better equipment. Therefore we consider it highly unlikely that this can explain the differences in performance.

A more serious concern relates to differences in age at implantation. While we tried to match the groups according to these criteria, good performers were on average implanted eleven months earlier than poor performers (at least when pair 9 was excluded from group comparisons). Therefore, it is possible that age at implantation had influenced cortical plasticity after CI implantation. To investigate the influence of age at implantation on observed effects, we included this factor in all subsequent steps of analysis. Age at implantation did not result in group differences or significant correlations for behavioral or for neurophysiological results. Consequently, differences in age at implantation–at least statistically- do not seem to be a likely explanation for the results of our study. Still, the current study population was–due to the limited number of suitable long term patients- rather small. It is possible, that an increased population size would have resulted in an effect of age implantation, but—obviously—the effect was so small that it demanded larger group sizes. As a consequence, we cannot rule out, that the differences in age at implantation led e.g. to weaker phoneme representations in the auditory cortex of the poor performer due to maladaptive cortical plasticity following the longer period of signal deprivation.

Therefore, unless other unknown reasons are responsible for the differences between groups, we are convinced that phoneme discrimination abilities have a strong influence on language development within the normal range in prelingually deafened CI users. Though lip reading or the interpretation of other visual information (such as sign language or gesturing to support language comprehension) is of great value in daily life communication and early on during rehabilitation, a profound cortical representation of phonemes is of utmost importance for good language comprehension. Therefore,—as shown here—testing phoneme pairs that are difficult to differentiate can serve as an effective predictor of language performance and of language function in general. Early and intensive training of phonological abilities should be established as a clinical routine in prelingually deafened CI users to strengthen cortical representation of phonemes in the auditory cortex. A training that is initially cross-modal, but that gradually reduces the visual input–in terms of shaping, for example–could be highly effective [78]. The resulting strengthening of the cortical representation should then facilitate higher levels of language processing, leading to enhanced language performance. Taken together, our results show that very different brain signatures can be evoked by the physically same stimuli depending on the context in which they appear and on how well the hearer developed after implantation. Even more advanced technology seems to be no guarantee for the high outcome of language functions. Stressing the role of cortical plasticity, one of the major challenges for future research is to develop individual training programs that allow each CI user to gain as much benefit from the signal provided by the implant as possible. As already pointed out by [22], we will only know the individual limitations in performance after “optimized, long-term training” (p. 690).

## Supporting Information

S1 Fig Stimulus /bu/. (TIF)

S1 Table Criteria for speech and language performance. (PDF)

S2 Table Results of speech intelligibility tests. (PDF)

S3 Table Subjective rating of hearing abilities. (PDF)
