# Supplementary figures and images for: When Hearing Is Tricky: Speech Processing Strategies in Prelingually Deafened Children and Adolescents with Cochlear Implants Having Good and Poor Speech Performance
Source: PLoS One. 2017 Jan 5;12(1):e0168655. doi: 10.1371/journal.pone.0168655 (PMC5215792; doi:10.1371/journal.pone.0168655)

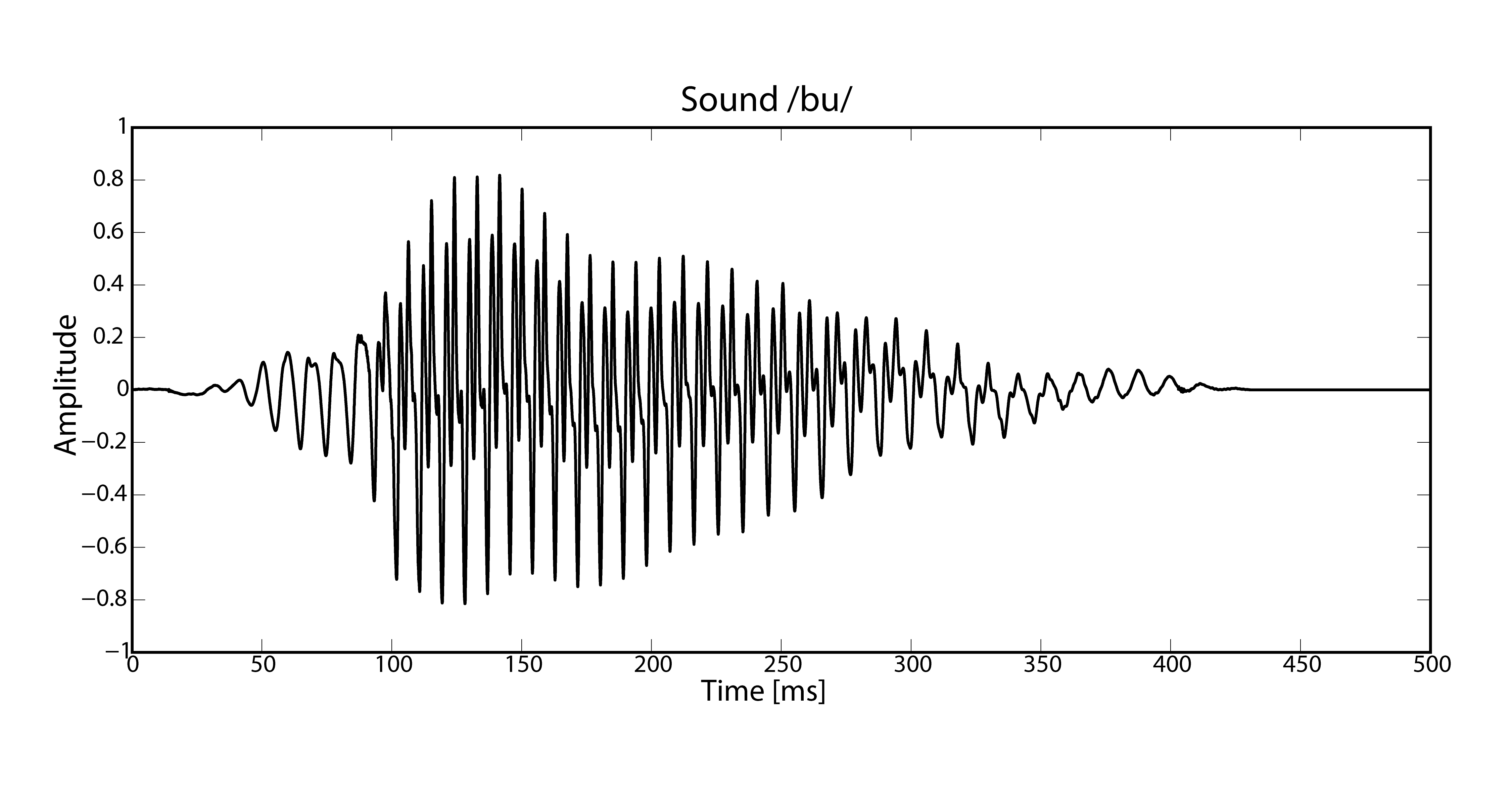

Supplement: S1 Fig — (TIF) [file pone.0168655.s001.tif]
