# Supplementary material for: When Hearing Is Tricky: Speech Processing Strategies in Prelingually Deafened Children and Adolescents with Cochlear Implants Having Good and Poor Speech Performance
Source: PLoS One. 2017 Jan 5;12(1):e0168655. doi: 10.1371/journal.pone.0168655 (PMC5215792; doi:10.1371/journal.pone.0168655)
Supplement: S1 Table — (PDF) [file pone.0168655.s002.pdf]

	**Good Performers**			**Bad Performers**		
**Assessment of speech/ language performance**	**1**	**2**	**3**	**4**	**5**	**6**
Articulation (based on Fox, 2003)	No conspicuousness; inconspicuous sound of voice in spontaneous speech (no noticeable nasality; prosody and pitch of speaking voice inconspicuous)	Minor phonological abnormalities (e.g. elisions of unstressed syllables); good intelligibility and inconspicuous sound of voice	Mildly impaired comprehensibility, phonological abnormalities (substitution of vowels or consonants; assimilations of consonants; reduction of syllables within words, elisions or addition of consonants) appear repeatedly and/or mild phonetic abnormalities (e.g. dyslalia)	Phonological errors appear frequently and/or 2-3 consonants cannot be formed and/or impaired vocal sound; comprehensibility is reduced	Incomplete phonemic inventory (conspicuous phonetic development) and/or many phonological errors and/or strongly modified sound of voice (e.g. nasality) that affects comprehensibility severely	Almost incomprehensible spontaneous speech
Syntactic and morphological skills (based on Clahsen, 1988)	Inconspicuous syntax (correct position of verbs in main- and subordinate clauses) and morphology (correct usage of morphological rules: gender-, case- and tense-marking; subject-verb congruency and plurals are used without errors)	Minor morphological abnormalities (e.g. impaired dative marking), syntactical rules are reliably mastered (correct position of verbs in main- and subordinate clauses)	Frequent morphological abnormalities (considerable difficulties in case marking, conspicuous subject-verb congruency); syntactical rules are generally mastered	Many morphological abnormalities and/or word order is not used systematically and/or reduced and conspicuous usage of function words (prepositions, conjugation, pronouns)	Many morphological abnormalities, unsystematic word order, strongly reduced usage of function words (prepositions, conjugation, pronouns)	Absent development of syntactic and morphological abnormalities, variable word order, no word inflection
Semantics and lexicon (based on Glück, 2011)	Very good development of lexicon; good and flexible ability to formulate; Test for Lexicon and Word Finding (WWT): Above average performance in the highest age-norms (t-value >60) or very good development according to hearing age (t-value 50-60)	Minor abnormalities in development of lexicon; WWT: good results in relation to age-norm from 10 to 12 years (t-value 40-60) or inconspicuous results according to hearing age (t-value: 50-40)	Abnormalities in vocabulary development; formulations without variations; WWT: conspicuous results in relation to age norm 8-10 years or conspicuous results according to hearing age (t-value < 40)	Reduced vocabulary development for content- and function words, strong abnormalities for low-frequency words; WWT: conspicuous results (t-value < 40 in the age norm 6-8 years) or significantly below average in hearing age adjusted norms	Only usage of high frequent function and content words	Rudimentary vocabulary; Almost exclusive use of nouns

Receptive language (based on Fox, 2011)	Inconspicuous TROG-D: highly developed comprehension of case markings and complex sentences	Minor difficulties in language comprehension, only slightly noticeable during conversation. TROG-D: able to comprehend complex sentence structures, understanding of personal pronouns in accusative and dative and double-object-constructions are possible	Mild difficulties in language comprehension, TROG-D: able to comprehend passive sentences and simple sentences	Frequent difficulties in language comprehension TROG-D: able to comprehend longer main clauses and dependent clauses in first position (because-sentences)	Highly impaired in language comprehension; able to comprehend simple SVO sentences	Comprehension of single words; meaning is deducted from context; unable to comprehend conversations about topics not grounded in the current situation

References:

Clahsen H. Normale und gestörte Kindersprache. Linguistische Untersuchungen zum Erwerb von Syntax und Morphologie. Amsterdam: 1988.

Fox A V. Kindliche Aussprachestörungen - Phonologischer Erwerb, Differenzdiagnostik, Therapie. 5th ed. Schulz-Kirchner; 2003.

Fox A V. TROG-D - Test zur Überprüfung des Grammatikverständnisses. 5th ed. Schulz-Kirchner; 2011.

Glück C W. Wortschatz- und Wortfindungstest für 6- bis 10-Jährige. 2nd ed. Urban & Fischer Verlag/Elsevier GmbH; 2011.
