# Supplementary material for: When Hearing Is Tricky: Speech Processing Strategies in Prelingually Deafened Children and Adolescents with Cochlear Implants Having Good and Poor Speech Performance
Source: PLoS One. 2017 Jan 5;12(1):e0168655. doi: 10.1371/journal.pone.0168655 (PMC5215792; doi:10.1371/journal.pone.0168655)
Supplement: S2 Table — (PDF) [file pone.0168655.s003.pdf]

Table 2: Speech intelligibility						
		FMWT		MedEL Teen- Ears Test Battery		
**Pair**	**Group**	**Word recognition (% correct)**	**Stimulus intensity (dB)**	**# of syllables (% correct)**	**Sentences in closed set (% correct)**	**Sentences in open set (% correct)**
1	GP	95	85	100	100	100
	BP	75	85	100	100	90
2	GP	90	75	100	100	100
	BP	70	75	100	100	85
3	GP	100	65	100	100	100
	BP	70	75	100	100	90
4	GP	100	75	100	100	100
	BP	80	75	100	100	100
5	GP	100	75	100	100	100
	BP	75	85	100	100	90
6	GP	100	85	100	100	100
	BP	85	85	100	100	70
7	GP	100	75	100	100	100
	BP	70	75	100	100	80
8	GP	100	75	100	100	100
	BP	85	75	100	100	95
9*	GP	70	65	100	100	95
	BP	80	85	100	100	85
GP:<br>Mean (SD):<br>Mean (SD) without pair 9:		95 (9.4)<br>98.1 (9.8)		100 (0)<br>100 (0)	100 (0)<br>100 (0)	99.4 (1.57)<br>100 (0)
BP:<br>Mean (SD):<br>Mean (SD) without pair 9:		76.7 (5.8)<br>76.3 (5.99)		100 (0)<br>100 (0)	100 (0)<br>100 (0)	87.2 (8.2)<br>87.5 (8.66)

Legend: Speech intelligibility in the Freiburg Monosyllable Word Test (FMWT) and the three subtests of the Teen-Ears Test Battery. Results show that basic speech intelligibility was good to very good in both groups and across all four tests. The FMWT was assessed at each participant’s most comfortable intensity (dB). Please note that stimulus intensity during EEG measurement and phoneme differentiation task was also defined via the most comfortable sound level.
