# Supplementary material for: When Hearing Is Tricky: Speech Processing Strategies in Prelingually Deafened Children and Adolescents with Cochlear Implants Having Good and Poor Speech Performance
Source: PLoS One. 2017 Jan 5;12(1):e0168655. doi: 10.1371/journal.pone.0168655 (PMC5215792; doi:10.1371/journal.pone.0168655)
Supplement: S3 Table — (PDF) [file pone.0168655.s004.pdf]

# Subjective rating of hearing

(According to the Manchester Teens Questionnaire: Post-operative)

| | never | rarely ever | sometimes | often | always |
|---|---|---|---|---|---|
| Are you able to discriminate sounds with your CI only by listening? (E.g. a car, a phone, a door bell, a barking dog)? | | | | | |
| Do you understand family and friends only by listening? | | | | | |
| Do you understand unknown persons / strangers only by listening? | | | | | |
| Do you easily understand people in noisy surroundings? | | | | | |
| Do you understand what is said in a group? | | | | | |
| Are you able to take part in a conversation in a group? | | | | | |
| Do you feel at ease when talking to people that you do not know very well? | | | | | |
| Do you contact your friends and family on the phone? | | | | | |
| Do you contact unknown persons on the phone? | | | | | |
| Do you easily understand telephone talk? | | | | | |
| Do you like watching TV? | | | | | |
| Do you like listening to music? | | | | | |
